# Editorial

**Published:** 1916-10

**Authors:** 


### EDITORIAL

The Atlanta Journal-Record of Medicine has its Business Office at 23 West Alabama Street where all business communications should be sent.

Remittances are payable to The Journal-Record of Medicine.

Reprints of Original Articles will be furnished at cost price. Requests for them had best be made on the Manuscript.

Upon request, we will present, postpaid, to each contributor of an original article, twenty copies of The Journal-Record of Medicine containing such article.

### THE SOUTHERN MEDICAL ASSOCIATION.

A little more than a year ago when the Fulton County Medical Society decided to invite the Southern Medical Association to meet in Atlanta, scarcely did we realize the benefit which we would derive from such a meeting, but now that the meeting is over we are able to realize even more fully how greatly we have benefited from this gathering in many ways. Any meeting together of men following similar lines of work where they may interchange experiences and ideas is of value and much more so when there are present those who have had such wide and varied experiences. The mere gathering together of the physicians and surgeons of the Southern States would have been helpful to these men, but when in addition, there are men present not only of national but international experience and reputation, it makes such a meeting the more valuable. From such a meeting we gain knowledge, but more important than this is the enthusiasm gained, the desire to go ahead and do better and more careful work.

Never has there been any movement started of so great importance to the Southern physician as the organization of the Southern Medical Association ten years ago. That this has been fully realized by the physicians of these states is shown by the interest taken in this Association and the very large number of men attending its meetings. Its growth has been steady and almost phenomenal. This, its tenth annual meeting, was by far the largest meeting ever held by the Association, both in the number of men present and in the character of papers and clinics. We think so because of the fact that we realize what the Southern Medical Association and its Journal mean to us. It is now second only to the American Medical Association in size and it is exceeded by none in the character and importance of papers read and clinics held. Each annual session really constitutes a post-graduate course in medicine, surgery and the various specialities.

To Dr. Seale Harris more than to any other one man belongs the credit for the growth and success of this Association. To Dr. Stewart Roberts, general chairman, and to the chairmen of the local committees we are indebted in a great measure for the success of this session in Atlanta. To these men we extend our gratitude for their untiring and successful efforts.

It will be impossible to mention all the clinics and papers which were of unusual interest at this meeting in Atlanta, but especial emphasis should, we think, be given the symposium on Cardio-vascular-renal diseases and the symposium on pellagra of the medical section and also the medical clinic of Dr. Barker on diseases relating to the disturbances of the internal secretions.

To the Southern Medical Association: We are glad you came, we were glad to have you with us on this, your tenth anniversary and shall expect you again ere another ten years elapse. We feel that we have been the gainers by your meeting with us. It is impossible for physicians of any city to have this organization meet with them and not be very greatly benefited thereby. A. H. B.

